# Understanding women’s and men’s perspectives on cervical cancer screening in Uganda: a qualitative study

**DOI:** 10.1186/s12885-024-12671-2

**Published:** 2024-08-01

**Authors:** Kathryn Bouskill, Glenn J. Wagner, Mahlet Gizaw, Joseph KB Matovu, Margrethe Juncker, Eve Namisango, Sylvia Nakami, Jolly Beyeza-Kashesya, Emmanuel Luyirika, Rhoda K. Wanyenze

**Affiliations:** 1 RAND Corporation, 1776 Main Street, Santa Monica, CA 90411 USA; 2 School of Public Health, Makerere University, Kampala, Uganda; 3 Faculty of Health Sciences, Busitema University, Mbale, Uganda; 4 Rays of Hope Hospice Jinja, Jinja, Uganda; 5 African Palliative Care Association, Kampala, Uganda; 6 Mulago Specialized Women and Neonatal Hospital, Kampala, Uganda; 7 School of Medicine, Makerere University, Kampala, Uganda; 8 Frederick S. Pardee RAND Graduate School, RAND Corporation, Santa Monica, CA USA

**Keywords:** Cervical cancer screening, Barriers, Facilitators, Social beliefs, Spousal support, Uganda

## Abstract

**Background:**

Cervical cancer remains a significant but preventable threat to women’s health throughout much of the developing world, including Uganda. Cervical cancer screening and timely treatment of pre-cancerous lesions is a cost-effective means of mitigating cervical cancer morbidity and mortality. However, only 5% of women in Uganda have ever been screened. Barriers to screening, such as social stigma and access to safe conditions, have been previously identified, but insights into the role of male spouses in encouraging or discouraging screening have been limited. To our knowledge, no studies have compared barriers and facilitators among women who had or had not yet been screened and male partners of screened and unscreened women.

**Methods:**

To resolve this gap, we conducted 7 focus groups– 3 among women who had been screened, 3 among those who had not been screened, and 1 among men whose female partners had or had not been screened. We performed qualitative thematic analysis on the focus group data.

**Results:**

We identified several important factors impacting screening and the decision to screen among women, ranging from stigma, availability of screening, false beliefs around the procedure and side effects, and the role of spousal support in screening promotion. Male spousal perspectives for screening ranged from full support to hesitancy around male-performed exams and possible prolonged periods without intercourse.

**Conclusion:**

This exploratory work demonstrates the importance of dialogue both among women and their male partners in enhancing screening uptake. Efforts to address screening uptake are necessary given that it is an important means of mitigating the burden of cervical cancer. Interventions along these lines need to take these barriers and facilitators into account in order to drive up demand for screening.

**Supplementary Information:**

The online version contains supplementary material available at 10.1186/s12885-024-12671-2.

## Background

Cervical cancer (CC) is a significant public health issue in Uganda, where the age-standardised incidence rate of 54.8 and age-standardised mortality rate of 40.5 per 100,000 are some of the highest in the world [1–4]. Cervical cancer accounts for around a quarter of all cancer deaths among Ugandan women [1] with 80% presenting with advanced disease (i.e., stage III or higher) [5]. 

Treatment for advanced CC in Uganda (outside of palliative care) remains limited to radiotherapeutic, chemotherapeutic, surgical provided through the Uganda Cancer Institute (UCI) in Kampala [5]. Due to the scarcity and high costs to accessing care, especially for those outside of the capital, [6] and prevention of advanced CC through screening is paramount.

Unfortunately, the lifetime screening rate for CC in Uganda is estimated to be as low as 5%, and the prevalence of human papilloma virus (HPV), which causes CC, is around 34% [5, 7]. Guideline recommendations call for CC screening every 3 years, but no formalised national effort has been mobilised [8]. 

In Uganda, CC screening is conducted with a visual inspection of the cervix with acetic acid (VIA) or pap smear [9]. Rapid HPV testing is emerging in Uganda, but is limited [10]. A systematic review of barriers to CC screening in Sub-Saharan Africa identified a range of significant barriers: fear of screening and embarrassment, fear of a positive screen, lack of privacy during screening, social stigma, lack of access and prohibitively high costs, negative experiences with health care personnel and at health care facilities, and a lack of spousal support [11]. Similar findings were found in a systematic review of barriers and facilitators to screening in Uganda [6]. The procedure is inexpensive if it is not free where it is available, but access outside the capital Kampala is limited, and the vast majority of the Ugandan population is in rural areas. Removal of pre-cancerous lesions is performed through thermal therapy, and at no or low cost, but is also not widely available outside of Kampala.

Qualitative explorations of women’s experiences with CC screening in Uganda have revealed additional barriers to screening, including lack of awareness of CC, misconceptions such as CC being attributed to contraceptive materials and witchcraft, fear of unsafe conditions from unsterilised screening instruments, and confusion over the purpose of asymptomatic screening [12–14]. These barriers, such as lack of awareness, cost of screening, distance to be screened, embarrassment, and lack of spousal support, were echoed in a systematic review of barriers to CC screening in low- and middle-income countries [15]. Another recent systematic review of drivers of CC screening in East Africa confirmed the aforementioned factors, as well as interpersonal stigma at the community level [16]. 

There has been recent exploration of the importance role of spousal support in the use of CC screening in Sub-Saharan Africa, and particularly in Uganda. Rawat et al.’s study in rural Uganda of men’s knowledge, beliefs, and perspectives called attention to the cultural importance of men’s power in household decision-making, including whether or not women are permitted to attend cervical cancer screening, highlighting the critical importance of involving men in the implementation and education of community-based cervical cancer screening programs [17]. An earlier qualitative study found concerns from Ugandan women that their spouse might think they were unfaithful if they engage in CC screening [18]. Studies have pointed to the positive impact that spousal support can have on intent to screen, [19, 20] while lack of emotional and financial support from a spouse and fear that a spouse would leave a woman if she were found to be at risk for CC are barriers to screening [12, 21]. Another study found that women would be willing to engage in screening irrespective of their spouse’s view [22]. These preliminary findings of the role of spousal influence over screening warrant the need to continue highlighting the importance of improving men’s support for CC screening and women’s empowerment into interventions and programs to increase CC screening uptake, [11] as well as push to engage local community women leaders to promote screening [6]. 

As part of the development of a peer advocacy intervention to promote CC screening, [23, 24] we conducted a series of focus group discussions with Ugandan women who had or had not screened for CC, and with men whose wives had or had not been screened. This work contributes an understanding of women’s experiences of being screened and their readiness to serve as advocates for other women to be screened, the ways in which men’s perspectives on screening can come to bear on whether or not women are screened, and the persistence of awareness of and access to screening as critical drivers of screening uptake.

## Methods

### Study design

This paper presents findings from the qualitative research conducted in the initial phase of a larger study that conducted a pilot randomised-controlled trial of a peer-led, group intervention aimed at training women who had screened for CC to conduct advocacy for CC screening among women in their social networks [23, 24]. This first phase consisted of a series of seven focus group discussions– three with women who had been screened (and in some cases, treated for cervical cancer risk) for CC, three with women who had never been screened for CC, and one with men whose spouses had either screened or not for CC. The study protocol was approved by the [redacted], and [redacted].

### Sample and location

Participant recruitment and focus group discussions took place at Buyinja Health Centre in Namayingo, a rural community in the Busogo region of Uganda. CC screening and thermal therapy are available at Buyinja Health Centre, as well as another nearby health centre in the district (Banda Health Centre), and from Rays of Hope Hospice Jinja (RHHJ), which conducts periodic mobile CC screening and thermal therapy “day camps.” Women who need biopsies are sent to Jinja (approximately 90 km from Namayingo), and if cancerous lesions are present, they are referred to the Uganda Cancer Institute, the tertiary public cancer care centre located in Kampala.

### Recruitment

To assess the range of sentiment towards CC screening, we sought to recruit women between ages 18–34 and over 35 who had been screened, women between ages 18–34 and over 35 who had not been screened, and men aged 18 + whose partners had and had not been screened. RHHJ maintains a registry of women who they have screened for CC, which was used to identify women who had screened within the past year. An RHHJ community health worker used the registry to purposively sample from a random list of women who had screened negative and screened positive (and received treatment if pre-cancerous lesions were present); these women were informed of the study and then, if interested in participating, were linked to the study coordinator for consent procedures. The women who had not been screened and men whose partners had and had not been screened and treated were also identified by RHHJ community health workers and contacted via phone. Written informed consent was given by all participants. We recruited a convenience sample of 50 women roughly equivalently split across those who had or had not been screened, and stratified into younger (18–34 years) and older age groups (35 + years). Men were recruited through staff and their network through local community health centers. This led to an additional 8 men, 4 of whose wives had and 4 whose wives had not been screened, participated in the male focus group discussion. All participants received 25,000 Ush (~$8 USD) for their participation.

### **Data collection instrument and procedures**

The women’s focus group discussion guide asked whether women discuss CC and CC screening, and if so, what is discussed and with whom. Specifically, any questions, fears, and concerns with screening were discussed, as well as factors that motivate discussions of CC screening. The men’s focus group discussion guide asked about perspectives on their wives’ health, their knowledge of screening and whether they know anyone who had been screened, perspectives on women’s need to abstain from sexual intercourse following treatment for cervical lesions, and opinions about the proposed intervention. Both focus group discussion guides were translated from English into Lusoga or Lusamia (depending on the tribal composition in the area where the focus group discussions were conducted) and reviewed by the study team for accuracy. The women’s focus groups were facilitated by skilled Ugandan women moderators, while a skilled Ugandan male moderator led the men’s focus group. The group discussions, each of which were over two hours, were conducted in Lusamia or Lusoga, audio-recorded, translated into English, transcribed and then uploaded into the qualitative data analysis software, Dedoose [25]. 

### Qualitative data analysis

The team created a codebook that mapped onto the respective focus group discussion guides and represented various domains of interest (e.g., whether and how people discuss CC screening; misconceptions of CC and CC screening; sources of stigma). We began by double-coding two transcripts, and then calculated interrater reliability across the all transcripts with a pooled Cohen’s Kappa coefficient for each of the codes. Coding procedures were discussed and revised until the pooled Cohen’s Kappa coefficient was > 0.80, which indicates near-perfect agreement among a coding team [26]. Any discrepancies were resolved through discussion. We followed standard approaches to identify key themes, or the range of responses under each code, by noting specific words, phrases, and ideas [27]. We identified additional themes through repetition and, metaphors used, and through existing literature of sources of CC-related perceptions [28]. 

## Results

### Overall impressions of cervical cancer screening

The seven focus groups– three with women who had been screened for CC (*n* = 35), three with women who had not yet been screened (*n* = 15), and one focus group with 8 men (4 of whom had wives who had screened and 4 whose wives had not)– revealed a general awareness of CC and relative openness to sharing one’s status of having been screened (and in some cases treated for CC-related lesions).

**Table 1:** Focus group participants

Focus Group Participant Type	Age Range	Number of total Participants
Screened Women	18–34	19
Screened Women	≥ 35	16
**Unscreened Women**	**18–34**	**8**
**Unscreened Women**	**≥ 35**	**7**
**Male Partners whose female partners had screened**	**≥ 18**	**4**
**Male Partners whose female partners had screened**	**≥ 18**	**4**

No clear differences were observed across the groups of younger and older women, and misconceptions and perceived stigma were present among those who had and had not been screened. Women who had not been screened were open to screening, but cited barriers such as lack of availability, lack of awareness of when screening is taking place, prohibitive cost, mistrust from their partner and adverse impacts on their relationships, and fear of pain, discomfort, and stigma from screening. Some were unaware of CC and that it can be prevented when detected early. Several misconceptions of CC screening and CC more generally were also reported by women who had been screened. Some men were motivated to support screening and treatment to relieve their partners of symptoms and avoid treatment-related costs and abstinence, while others expressed concern over having women be screened by other men.

Most women who had been screened spoke about CC screening and treatment with friends, church parishioners, sisters, mothers, co-wives– but they reported often neglecting to follow up with their peers after initial discussions of screening. Nearly everyone who had screened emphasized its importance and noted that it may be uncomfortable, but not painful. Others described how local nurses promote screening, even “mobilising us even during funeral services and burials.” Despite this relative openness, participants also shared misconceptions and barriers to getting screened. It is also important to note that some unscreened women complained of severe symptoms that are often indicative of later-stage CC, such as back and pelvic pain and vaginal discharge. For example, one older unscreened woman noted, “I have prickly pain inside my private parts. I no longer have sex with my husband. I cannot bear the pain” (unscreened 35 + woman). Only women who had been screened and treated for pre-cancerous lesions discussed ways to prevent CC, and all spoke favourably about their experience of reducing their CC risk.

### Women’s impressions of cervical cancer screening and treatment

#### Promoting screening and preventing infection

All women spoke of peers who have questions about the screening procedure, and screened women reported that they would use their experience to inform unscreened women about what to expect. A screened woman explained,

I asked my friend if she has ever heard about anything concerning CC screening, and I explained to her that she will be asked to go into a private room, they will ask her name, then she will be told to remove her undergarments and then sit in a position that requires her to open her legs. A machine will be fixed into her private parts and if it dictates that she has CC risk, medicine will be inserted in her there, and then but if she does not have CC the machine will be removed. I even told her that her uterus will not be removed like many people assume (screened 18–34 woman).

This quote highlights an additional misconception and concern among women that the uterus will be removed during screening, which will be described in more detail below.

Women who had been screened for CC spoke of the need for women to visit a health facility and be screened if they are experiencing symptoms. One screened woman explained,

As women, we tend to meet and complain a lot about our health…Go to the hospital and screen so that they tell you what exactly is affecting your health. You may have or be developing CC. Don’t complain while sitting at home talking to people who won’t help you. You still have a lot of responsibility and roles to play in your homes. You have children to up bring, home to look after, and gardens to tend, but above all you need life, and the only way is by going to the hospital (screened 35 + woman).

Screened women cautioned others about the need for continuous periodic screening and other preventative measures, such as using condoms before one is married, “I do tell my friends that every time there is an opportunity to screen for CC they should do it because our men keep having sex with multiple partners, so we can never be sure that we are safe. You may think that the CC is cured completely and yet the man re-infect you. Men should also go for circumcision and keep clean” (screened 18–34 woman).

Another added, “I will give an example of us women who are not yet married, a man would want to have unprotected sex with us which may lead to CC. I think it is good practice to use a condom especially for us who are not married to reduce the risk of contracting CC.” (screened 18–34 woman).

### Misconceptions about cervical cancer screening

Misconceptions related to CC were reported by both screened and unscreened women. As noted, one of the primary misconceptions is that screening involves removal of the uterus. Women who had undergone screening dispelled this myth, as exemplified in this quote from a screened woman, “Yes, when people talk about screening, they say that they pull out the uterus and put it on the table to have it checked, and it makes them change their mind about screening” (screened 18–34 woman). However, this false belief was echoed by several unscreened women, some who believed the uterus could be reinserted upside down, leaving a woman barren.

Another screened woman described having to dispel a rumor that screening involves “roasting the uterus,” assuring her friends that the procedure is “not so painful, but you have to ensure the whole process if you want to know and be treated” (screened 18–34 woman). Still, several unscreened women had similar sentiments: “They say those machines they use to examine the cervix are pushed inside deeply and so intense is the pain. This has scared off so many of us. But, I feel I need to put away the fear and come today to see or get the experience myself simply because much as they scare us, many are testing and getting their life back. Yet we are left behind dying slowly but surely” (unscreened 35 + woman).

Other women who had been screened said nurses had told them that genital hygiene or having sexual intercourse during one’s menstrual period can lead to CC. Another woman noted, “The nurses advised us not to use our long nails to wash inside our private parts because it may also cause CC,” or that “dirt in the knickers causes CC” (screened 18–34 woman). In addition, one woman who had been screened also noted, “I overheard people say that syphilis is also symptom of CC” (screened 18–34 woman).

The concern that contraceptives cause CC was also shared by women who had been screened for CC. However, it is possible that women were conflating the risk of CC with having multiple sexual partners. One woman who had not yet been screened spoke of telling her husband they could no longer use condoms because they would cause CC. Another screened woman noted, “I would tell my friend that in case she uses family planning she will get CC, and also if she has sex with more than three men, she is most likely going to get CC” (screened 18–34 woman). An unscreened woman (35+) said, “I have never seen anybody die from being screened. I also dismissed that story. I need to be screened.”

Another important misconception was that screening and treatment can stretch out a woman’s genitals and make her “undesirable” to her partner. One screened woman said, “We get so many and funny questions. One asked me whether that screening machine doesn’t enlarge or stretch one’s private parts and become too wide for her husband to enjoy her tightness during sex” (screened 35 + woman), to which another answered:

I tell them that it’s not even the size of the head of the baby, and not even bigger than the normal penis which goes there every other day, so you tell her the reality and the truth about that. One asked me how the lesions are burned and if there is pain. I told them all you feel is the rod being pushed in but the rest is not felt. There is nothing like a burning sensation. It’s a small rod which is inserted but no pain at all. Their fears are around that; they are scared that once they burn the lesions, they will instead widen and spread into the uterus…They believe once a cancer is burned they think it never heals. But I tell them my story. I am a living testimony. I was treated with that procedure and I am now normal (screened 35 + woman).

In addition to these misconceptions, women who had not been screened described additional structural barriers to getting screened, which are described below.

### Additional barriers to getting screened

#### Cost of screening

Although VIA screening is supposedly free of cost, women also cited screening-related costs. It may be a misconception that women believe there are costs to being screening, but transportation, loss of work time, and treatment can bring about costs for women and their families. One unscreened woman said, “What I usually tell them wherever I am, wherever I get these various pains is how I feel and the pains I go through but what is causing these pains is because I never got screened, not even treated to know what I am suffering from. I tell them that this all due to poverty. How I wish I could afford I would go, get screened and know what is causing all this and get treated” (unscreened 35 + woman).

### Stigma

Social stigma did not seem to play a major role in the challenges that women expressed regarding CC screening and treatment; nonetheless, some women did report experiences with social stigma and discrimination. Both the male and female participants in their respective focus groups expressed an openness in speaking about CC, but some women who had been screened and treated for pre-cancerous lesions reported getting laughed at and having people “point fingers” at them for living with cancer risk and also experiencing internalized stigma for their diagnosis. Another unscreened woman was concerned about gossip in her community if the screening revealed she had pre-cancerous lesions, “I do fear somehow because I may trust some person and confide in her, yet this person is going to betray my trust and go on telling the whole community about my status. Before long everybody will have known” (unscreened 18–34 woman). Another woman who had been screened described the process of getting undressed and being examined as “shameful,” but still advocated that others be screened to stop pre-cancerous lesions before they become symptomatic.

Furthermore, some were mocked when talking about health risks when they are not health care workers themselves. As one woman recounted, “Sometimes when we talk to people about going for screening, they say, ‘Are you a health worker?’ As in, why would I have the audacity to come and start teaching them about CC” (screened 18–34 woman). Hence, women described the importance of credibility when discussing CC screening.

### Fear of adverse impacts on intimate relationships

As noted above, others feared telling their husbands that they would have to abstain from sexual intercourse for several weeks following treatment, adding, “I fear to disclose at times because the health workers tell you not to have sexual intercourse for six weeks, some men cannot accept you to deny them sex to wait” (screened 18–34 woman). Another woman who had not been screened stated, “Honestly, I fear to be screened by a man I would prefer a fellow woman to screen me…It looks shaming especially to a man who is not my husband. I do not wish to be seen by a man” (unscreened 35 + woman).

Other women were able to overcome their husbands’ resistance. One screened woman spoke about this challenge:

I tell my friends that even when you feel much better you should not have sex for a period of one month and one week or until complete your medication. I give an example where my husband wanted to have sex with me when I was receiving treatment, and he said, “Do you want me to go have it with a goat?” and I comforted him and told him that its better we follow the orders given to us by the nurses and better to take free medication rather than you having to spend a lot of money on me when the CC reappears. My husband insisted that maybe I have got another man with whom I am having sex, but I gave him the phone number of the nurses for him to be able to witness for himself that it is the nurses who gave me those instructions (screened 18–34 woman).

Women who had been screened and treated spoke unanimously about returning to good health and normal sexual relations after being treated, which could allay concerns on the part of their husbands. One woman added, “I talk about the trouble I went through [before being treated]. I had stopped having sex with my husband completely. I had so much pain– backaches and lower abdominal pain. The moment I would have sex, I would yell and ask him why he is not ending this painful game, but now all that is no more. I tell them I got healed of all the sign and symptoms. I am normal now” (screened 18–34 woman).

Screened women also described initiating conversations with wives of men whom they had identified as risky: “I started initiating the conversation with my sister-in-law as I realized that her husband is a womanizer and is HIV positive, and women living with HIV are at a high risk of getting CC” (screened 18–34 woman). Another added, “I intend to talk to my husband because he is the kind of man who sleeps around with every woman he admires” (screened 18–34 woman).

### Motivation to be screened

Women who had not yet been screened mentioned becoming motivated to be screened after they were educated by others about the procedure and the benefits of CC screening during the process of participating in a focus group. Women who were symptomatic learned that CC can be “cured” if treated early, even though they had not yet been screened. One younger woman said,

What gives me the courage to talk about cervical cancer is once I am screened and treated, I will become a living testimony…Even if I am negative, I will become a champion who will speak from a point of a person who understands the disease due to the education I will have got. I need to empower my friends with my experience and the need to tell them of the progress I have made in whatever way. If not, I will preach the gospel of healing. The possibility to heal gives me the courage to talk (unscreened 18–34 woman).

This was particularly poignant for women who were experiencing severe symptoms, with one older woman who had not yet been screened recounting, “I talk mainly about the possibility of getting treatment because what I know is that once I am treated, I will be able to live my full life again. I no longer work. I have lots of pain now all over my private parts. What I need is treatment. So, I will keep inquiring about when the doctors will be back and I will go for screening” (unscreened 35 + woman). This led women to be more open to spreading the message that screening and early treatment can prevent cancer, although there were still misconceptions regarding pre-cancerous lesions. As one woman noted, “The belief I had that cancer is not curable are now gone because of joining the meeting today. Such feelings are now gone. I know the truth so I want to be an ambassador who will tell people that” (unscreened 18–34 woman).

### Overview of men’s perspectives

The male focus group was comprised of men whose wives had been screened and those whose wives had not. Acceptance of screening, support for their wives, and misconceptions differed within the group. Several men lamented that it was “ignorance” among men and women that was causing women to die of CC and championed the need to educate those in Namayingo about CC screening and treatment. However, others expressed misgivings with having their wives be screened by men and not being able to have intercourse for a short period of time if pre-cancerous lesions are removed.

### Women’s symptoms as motivation to encourage screening

All men who had witnessed a positive screen and treatment were in favor of screening initiatives. One man noted, “I just feel good– what can I say? My partner got healed. There is a benefit to mobilising other women to get screened, treated, and healed.” A few men were able to describe the process of screening and treatment, including temporary abstinence from sexual intercourse, in detail. One man assured the other men that it was because of the screening and the treatment that his wife received that, “she is now fine and got healed.” Another described the challenges of having his wife experience pain during sexual intercourse, adding, “when she is screened early and treated, she’ll get healed. I witnessed this when my own wife got healed. That is [the] reason why I want her to be screened again to avoid problems which might make me not eat good sauce [have sex].” These testimonies led other men to react in saying, “My wife has not been screened yet, but after what I am hearing here, I have to take her to be screened,” before adding that access to health care services is limited across the board. Another, whose wife was unable to have sex because of pain and was treated, pleaded for more “machines that test for cervical cancer because we are going to bury someone who died of CC.”

### Financial concerns as motivation to encourage screening

One man described how he sent his wife back to her parents’ home and accused her of being a drain on his finances following her diagnosis. Then, after facing condemnation from his wife’s parents, he took her back and she started treatment and is now “very fine.” The man had then started to “advocate for better CC screening and treatment at our health center.” Men also spoke of the respect for their wives as “mothers of the nation,” and how “if a woman acquires CC, it means we men also get a shock.” Another added that he knew of the shock and the difficulty of having to allocate two months of wages to his wife’s treatment.

One man described having his second wife also get screened, and the relief he felt when she was not found to have any lesions because he was “financially handicapped” from treating his first wife. Other men echoed the financial hardship of treating advanced CC. One man described his sister-in-law’s experience, adding, “She consumed all our money in the struggle to cure her cancer, but now she is looking very strong and healed.”

### Men’s mistrust over the screening procedure and screening-related abstinence

Some men described having mistrust in their partner and frustration over not being able to have sexual intercourse if pre-cancerous lesions are found and removed following screening. Following a diagnosis, one man said,

It brought serious problems and misunderstanding between me and her. I told my wife that if it is like this then you better go back to your parents’ home because I can’t stay with a person without doing “the work I do” (having sexual intercourse). She explained her issues to me privately. I said, “you are lying me. Why haven’t you been telling me?” She replied, “I have been waiting to tell you, but I was afraid.” I said I would take her to the doctor to prove whether she is telling me the truth.

Others carried the misconception and mistrust of men performing screening and treatment on their wives, reporting rumors of a doctor who has a “bad habit of having sex with his patients.” Another added,

A male health worker enters the room with my wife and they close the door. Even if the health workers didn’t have any thoughts of an affair, I as a man am still in fear…You can’t know whether the health worker is having sex with your wife. Health workers are human beings– they are not bishops! Therefore, only female health workers should work on fellow women in the cancer department.

Others refuted this worry, adding that they do not have sex with female nurses when they are alone with them being treated, so men should be able to screen the cervix, and that male doctors would not want to contract the virus, adding, “a health worker is just medical personnel regardless of the gender.”

Men also had several questions regarding CC and reproductive health more broadly, including whether “CC” [HPV] could be transmitted to a male partner, whether circumcision impacts transmission, how cancer spreads, why men cannot be screened, how CC impacts pregnancy, and whether there are male doctors who perform screenings but do not have wives (and may try to have sexual intercourse with their wives).

Men also conveyed several misconceptions with respect to CC risk. Like some of the female participants, one man talked about a previous belief that witchcraft causes CC. Another mentioned that he had learned that family planning can cause CC. Others spoke about how pain during childbirth or giving birth to a stillborn child can cause CC. Another cautioned that screening and treatment can cause the removal of the uterus.

## Discussion

The themes from these focus groups revealed support for social influences to encourage women to get screened (and treated) for CC risk, but also important insights into how misconceptions, stigma, and fears impede uptake of screening and treatment. Women who had not been screened described concerns over undressing in front of medical personnel and of negative impacts on their marriages, gossip within their social networks if they screen positive, and fear of becoming infertile (e.g., through the misconception that the uterus is removed during screening) through screening and treatment. Overcoming these issues is critical to increasing Uganda’s very low screening rates.

Despite these barriers, a predominant and encouraging theme among women who had been screened for CC was the desire and need to share their screening experience and to encourage peers to also get screened. Such sentiments were often conveyed passionately, particularly among women who had received treatment (either for pre-cancerous or cancerous lesions) and had their health restored. This highlights how women who have been screened can act as change agents for promoting CC screening, which was corroborated by a recent effective pilot peer-based intervention in Uganda [24]. The potential impact and power of social influence for dissemination of knowledge about CC by women with experience of CC screening was demonstrated even in the focus group discussions. Women who have never been screened spoke of having newfound revelations about the ability of CC risk to be cured through screening and treatment, and how this now motivated them to get screened. Having credibility from medical personnel who can attest to the importance and the fact that often fellow women perform the exams is important in increasing the motivation to be screened.

The women and men who participated in these focus groups revealed certain misconceptions, sources of stigma, and other challenges related to CC screening and subsequent treatment that are important to consider in both care delivery and in interventions to mitigate CC through increased uptake of screening (and treatment when needed). Informing women of the ability of screening and treatment to resolve physical symptoms, particularly if identified early, may help alleviate stigma and fear and thereby promote CC screening. In addition, Mwaka et al.’s 2018 research highlighted Northern Ugandan women’s worries over the removal of the uterus during cervical cancer treatment and the subsequent stigma from infertility. This furthermore underscores the need to inform women of the efficacy of early detection and treatment to avoid drastic measures like removal of the uterus [29]. 

A critical misconception expressed by some participants was that only symptomatic women should be screened for CC. If women wait for physical symptoms to be screened, the greater the likelihood that they will develop advanced signs of disease before these signs are detected and that treatment will be too costly to obtain. Indeed, the vast majority of Uganda women have advanced stage CC at first onset into care [30]. However, because treatment for CC is prohibitively expensive and difficult to access, increasing screening and concurrent treatment for pre-cancerous lesions is the most effective way to reduce the CC burden in Uganda.

Another misconception expressed by some participants was potential harm to the uterus. Given the fact that Uganda is a pronatalist nation where polygamy is common, being able to have children is paramount, and dispelling myths over infertility is critical. Women who had been screened spoke emphatically about how sharing their experience and knowledge can go a long way to dispelling such myths.

Including men’s perspectives was an important contribution that demonstrated the ways in which men can influence screening for CC. Several of the men spoke of the need for fellow men to support their women getting screened, and to even encourage other women in the community to get screened, in addition to fellow men to support their wives in getting screened. Hence, just as women can serve as agents of change, so too can men with regard to promoting women’s health. Women were exalted by some as “mothers of the nation” – to emphasize the importance of promoting the health and wellbeing of women in their community. However, the discussion also brought to light certain misconceptions and concerns, such as mistrust of male practitioners. This may be addressed by increasing the number of female practitioners who can deliver CC screening and treatment, or by inviting men to attend screening with their wives. Collectively, these findings and those described in Rawat et al.’s (2023) qualitative study of Ugandan men’s perspectives of CC screening underscore the critical importance of planning CC screening interventions that account for gender, power, and structural access to screening and women’s health more broadly [17]. 

This exploratory study is limited in terms of its external validity; i.e., it is unclear if the viewpoints presented here would hold across other regions of Uganda and East Africa more broadly. Given that CC remains a significant public health issue in Uganda, it is important to continue to gather context-rich qualitative data to identify ways to enact CC control. There is also a possibility that focus group participants already had more positive perceptions of CC screening and treatment given that they had already received care in health centers. There is also the possibility of social desirability bias whereby responses in the group may have been influenced by the presence of peers. Lastly, the current study does not directly take on the issue of availability and accessibility of screening, although this work shows that it can act as a significant barrier for women seeking to be screened. Despite these limitations, this study calls attention to the importance of understanding women’s interpersonal dynamics to influence CC screening, the role of men in women’s preventative health, and persistent barriers of access to and knowledge of screening and CC more broadly.

## Conclusion

Sentiment towards CC screening was largely positive, from both the female and male participants, demonstrating the need to augment access to screening and treatment performed by female health practitioners. Women who had been screened expressed a desire and willingness to openly share their experiences with other women, and to engage in advocacy for CC screening. The potential power of such advocacy was demonstrated in the focus groups themselves, as women who had never been screened expressed improved knowledge and motivation for screening because of their participation. However, stigma and misconceptions related to CC screening and treatment were present, which likely contributes to the low rates of screening. Interventions to control CC through timely screening and treatment will need to actively address these fears and misconceptions. Women sharing their personal experiences with screening and treatment may effectively address the need for dissemination of accurate CC-related information and stigma reduction. Furthermore, men can and should play an important role in CC screening interventions, both in terms of education and awareness, but also in dispelling misconceptions and stigma around male medical personnel. As is the case with women who have been screened, men whose wives have been screened should also be considered as important change agents for influencing other men to encourage their wives to get screened. Given that treatment for CC remains prohibitively expensive and difficult to access, improving screening rates is paramount for curbing the excessive burden of CC in Uganda and much of the developing world.

### Electronic supplementary material

Below is the link to the electronic supplementary material.

Supplementary Material 1

## Data Availability

The data collected include data that could potentially identify individuals participating in the study and could thus erode privacy of the participating individuals. We are, thus, unable to share the raw data, but will be willing to share the codebook and the focus group protocol if a reasonable request is made. Contact Glenn Wagner at gwagner@rand.org for enquiries.

## Abbreviations

CC: Cervical Cancer
HPV: Human Papilloma Virus
USh: Ugandan Shillings
USD: United States Dollar
RHHJ: Rays of Hope Hospice Jinja

## References

[1] African Cancer Registry Network [Internet]. 2018 [cited 2023 Dec 10]. Uganda - Kampala Cancer Registry. http://afcrn.org/membership/membership-list/81-kampala-uganda.

[2] Ferlay J, Ervik M, Lam F, Colombet M, Mery L, Piñeros M et al. Cancer today. 2018. Global Cancer Observatory: Cancer today. https://gco.iarc.fr/today/.

[3] International Agency of Cancer Registries [Internet]. 2018. Kampala Cancer Registry.

[4] Wabinga H, Parkin D, Nambooze S. Kampala Cancer Registry report for the period 2007–2009. Kampala, Uganda: Kampala Cancer Registry; 2012.

[5] Nakisige C, Schwartz M, Ndira AO. Cervical cancer screening and treatment in Uganda. Gynecologic Oncol Rep. 2017;20:37–40. 10.1016/j.gore.2017.01.009 PMC5331149 28275695

[6] Black E, Hyslop F, Richmond R. Barriers and facilitators to uptake of cervical cancer screening among women in Uganda: a systematic review. BMC Women’s Health. 2019;19(1):108. 31399092 10.1186/s12905-019-0809-z PMC6688246

[7] Ndejjo R, Mukama T, Musabyimana A, Musoke D. Uptake of Cervical Cancer Screening and Associated Factors among Women in Rural Uganda: A Cross Sectional Study. Tornesello ML, editor. PLoS ONE. 2016;11(2):e0149696. 10.1371/journal.pone.0149696 PMC4760951 26894270

[8] World Health Organization. WHO guidelines for the use of thermal ablation for cervical pre-cancer lesions [Internet]. Geneva: World Health Organization. 2019 [cited 2023 Dec 10]. 108 p. https://iris.who.int/handle/10665/329299. 31661202

[9] Auma J, Ndawula A, Ackers-Johnson J, Horder C, Seekles M, Kaul V, et al. Task-shifting for point-of-care cervical cancer prevention in low- and middle-income countries: a case study from Uganda. Front Public Health. 2023;11:1105559. 37575099 10.3389/fpubh.2023.1105559 PMC10420095

[10] Sultanov M, Van Der Schans J, Koot JAR, De Zeeuw J, Nakisige C, Greuter MJW, et al. EE685 screen-and-treat Cervical Cancer Screening Strategy using high-risk HPV testing: early evaluation for Uganda. Value Health. 2023;26(12):S186. 10.1016/j.jval.2023.09.950 PMC11414519 39302149

[11] Lim JNW, Ojo AA. Barriers to utilisation of cervical cancer screening in Sub Sahara Africa: a systematic review. Eur J Cancer Care. 2017;26(1):e12444. 10.1111/ecc.12444 26853214

[12] Ndejjo R, Mukama T, Kiguli J, Musoke D. Knowledge, facilitators and barriers to cervical cancer screening among women in Uganda: a qualitative study. BMJ Open. 2017;7(6):e016282. 28606908 10.1136/bmjopen-2017-016282 PMC5541520

[13] Natuhwera G, Ellis P, Acuda SW. Women’s lived experiences of advanced cervical cancer: a descriptive qualitative study. Int J Palliat Nurs. 2021;27(9):450–62. 34846937 10.12968/ijpn.2021.27.9.450

[14] Hasahya OT, Berggren V, Sematimba D, Nabirye RC, Kumakech E. Beliefs, perceptions and health-seeking behaviours in relation to cervical cancer: a qualitative study among women in Uganda following completion of an HPV vaccination campaign. Global Health Action. 2016;9(1):29336. 26895145 10.3402/gha.v9.29336 PMC4759844

[15] Srinath A, Van Merode F, Rao SV, Pavlova M. Barriers to cervical cancer and breast cancer screening uptake in low- and middle-income countries: a systematic review. Health Policy Plann. 2023;38(4):509–27. 10.1093/heapol/czac104 PMC10089064 36525529

[16] Atnafu DD, Khatri R, Assefa Y. Drivers of cervical cancer prevention and management in sub-saharan Africa: a qualitative synthesis of mixed studies. Health Res Policy Sys. 2024;22(1):21. 10.1186/s12961-023-01094-3 PMC10851545 38331830

[17] Rawat A, Mithani N, Sanders C, Namugosa R, Payne B, Mitchell-Foster S, et al. We shall tell them with love, inform them what we have Learnt and then allow them to go - men’s perspectives of self-collected cervical Cancer Screening in Rural Uganda: a qualitative Inquiry. J Canc Educ. 2023;38(2):618–24. 10.1007/s13187-022-02163-x 35384556

[18] Mutyaba T, Faxelid E, Mirembe F, Weiderpass E. Influences on uptake of reproductive health services in Nsangi community of Uganda and their implications for cervical cancer screening. Reprod Health. 2007;4(1):4. 17594474 10.1186/1742-4755-4-4 PMC1936416

[19] Twinomujuni C, Nuwaha F, Babirye JN. Understanding the Low Level of Cervical Cancer Screening in Masaka Uganda Using the ASE Model: A Community-Based Survey. Favato G, editor. PLoS ONE. 2015;10(6):e0128498. 10.1371/journal.pone.0128498 PMC4451264 26030869

[20] Paul P, Winkler JL, Bartolini RM, Penny ME, Huong TT, Nga LT, et al. Screen-and-treat Approach to Cervical Cancer Prevention using Visual Inspection with Acetic Acid and Cryotherapy: experiences, perceptions, and Beliefs from Demonstration projects in Peru, Uganda, and Vietnam. Oncologist. 2013;18(S2):6–12. 24334477 10.1634/theoncologist.18-S2-6

[21] Mwaka AD, Wabinga HR, Mayanja-Kizza H. Mind the gaps: a qualitative study of perceptions of healthcare professionals on challenges and proposed remedies for cervical cancer help-seeking in post conflict northern Uganda. BMC Fam Pract. 2013;14(1):193. 24341601 10.1186/1471-2296-14-193 PMC3915559

[22] Teng FF, Mitchell SM, Sekikubo M, Biryabarema C, Byamugisha JK, Steinberg M, et al. Understanding the role of embarrassment in gynaecological screening: a qualitative study from the ASPIRE cervical cancer screening project in Uganda. BMJ Open. 2014;4(4):e004783. 24727360 10.1136/bmjopen-2014-004783 PMC3987737

[23] Wanyenze RK, Matovu JKB, Bouskill K, Juncker M, Namisango E, Nakami S, et al. Social network-based group intervention to promote uptake of cervical cancer screening in Uganda: study protocol for a pilot randomized controlled trial. Pilot Feasibility Stud. 2022;8(1):247. 36476609 10.1186/s40814-022-01211-z PMC9727870

[24] Wagner GJ, Matovu JKB, Juncker M, Namisango E, Bouskill K, Nakami S, et al. Effects of a peer advocacy intervention on cervical cancer screening among social network members: results of a randomized controlled trial in Uganda. J Behav Med. 2023;46(6):930–9. 37702912 10.1007/s10865-023-00418-6 PMC10577098

[25] Dedoose. Los Angeles CA, SocioCultural Research Consultants LLC. 2016. (Web application for managing, analyzing, and presenting qualitative and mixed method research data).

[26] Burla L, Knierim B, Barth J, Liewald K, Duetz M, Abel T. From text to codings: Intercoder Reliability Assessment in qualitative content analysis. Nurs Res. 2008;57(2):113–7. 18347483 10.1097/01.NNR.0000313482.33917.7d

[27] Butler-Kisber L, Qualitative Inquiry. Thematic, Narrative and Arts-Informed Perspectives [Internet]. 1 Oliver’s Yard, 55 City Road London EC1Y 1SP: SAGE Publications, Inc.; 2010 [cited 2023 Dec 10]. https://methods.sagepub.com/book/qualitative-inquiry-thematic-narrative-and-arts-informed-perspectives.

[28] Ryan GW, Bernard HR. Techniques to identify themes. Field Methods. 2003;15(1):85–109. 10.1177/1525822X02239569

[29] Mwaka AD, Okello ES, Wabinga H. Perceptions and beliefs of lay people from northern Uganda regarding surgery for diagnosis and treatment of cervical cancer. Psycho-oncology. 2018;27(8):1965–70. 29719940 10.1002/pon.4751

[30] Mwaka AD, Garimoi CO, Were EM, Roland M, Wabinga H, Lyratzopoulos G. Social, demographic and healthcare factors associated with stage at diagnosis of cervical cancer: cross-sectional study in a tertiary hospital in Northern Uganda. BMJ Open. 2016;6(1):e007690. 26801459 10.1136/bmjopen-2015-007690 PMC4735146

