# Supplementary material for: Understanding women’s and men’s perspectives on cervical cancer screening in Uganda: a qualitative study
Source: BMC Cancer. 2024 Aug 1;24:933. doi: 10.1186/s12885-024-12671-2 (PMC11293159; doi:10.1186/s12885-024-12671-2)
Supplement: Supplementary file 1 — Supplementary Material 1 [file 12885_2024_12671_MOESM1_ESM.docx]

**Focus Group Guides**

The WLCCR groups will focus on barriers, facilitators and motivators of engagement in CC-related advocacy; how decisions are made about whether, when and how to bring up CC or to encourage someone to engage in CC protective behaviors; and eliciting feedback on the planned intervention (e.g., should facilitators also be WLCCR? Should workshops be specific to age ranges or with women of mixed ages?) The alter groups will discuss experiences in which others encouraged them or they encouraged others to seek CC screening or treatment, and what influenced whether such advocacy worked.

Game Changers: Women Living with Cervical Cancer Risk (WLCCR) Focus Group Protocol

Notes: Record eligibility information and go through informed consent prior to starting focus group and digital recorder. If any participant in the group declines to be recorded, the assistant facilitator should take verbatim notes and ensure that the focus group is not digitally recorded.

Thank you for coming here today. As I mentioned to you when you arrived, Buyinja Health Centre IV is working on a program to help people women who are at risk for developing cervical cancer educate people who they know (their family, friends, and others) about cervical cancer risk. You are being asked to participate in a group discussion to help design this program.

First I’d like to make sure we all agree on some ground rules:

Everyone should participate

One person speaks at a time

No side conversations

Ask if you don’t understand a question

No right or wrong answers

Answer honestly

Respect each other’s opinions

No mobile phones/texting

Speak up for recording

*Does anyone have any questions before we begin?*

| **Topic** | **Question(s)** |
| --- | --- |
| Grand Tour | 1. In general, how much do you talk with other women about getting tested for cervical cancer? How about your treatment for cervical lesions/treatment after being screened? |

| **Conversations about cervical cancer screening and treatment** | 1. Who do you talk to about cervical cancer screening? What about getting treated after your screening? (Probe for friends, other women in the community, women in your family, et al.) 2. What topics related to cervical cancer screening and treatment do you talk about? (probe for women’s gynecological health in general, risk reduction, testing, follow-up treatment) 3. Do you ever initiate these conversations about screening and treatment for cervical treatment? If so, why, and how do you decide whether to initiate these conversations? How do you start the conversation? With whom do you tend to initiate these conversations? How do you know when someone is open to hearing about cervical cancer screening? How about cervical cancer treatment? 4. [As a follow-up] Please tell me about a recent time when you initiated a conversation about cervical cancer screening; tell me who you talked with, what made you decide to initiate the conversation, what was said, and how the other person reacted. 5. Do other people ever initiate these conversations about cervical cancer screening with you? Do some people you know tend to initiate conversations more than others and if so, who? What kinds of questions do people ask you? (Probe for participant vs. specific others listed) 6. How do you react when other people talk to you about cervical cancer screening? How about treatment after screening? What kind of conversations lead you to change your attitudes around cervical cancer? Are some kinds of conversations more influential than others on your attitudes or behaviors? 7. What kinds of fears or concerns do you have about talking with other people you know about cervical cancer screening? How about treatment? (Probe for internalized stigma, self-acceptance of cervical cancer risk been screened and treated for cervical lesions to, experiences with in-network discrimination, level of disclosure, etc.) 8. What kinds of things motivate you or make you more comfortable talking with other people about cervical cancer screening? How about treatment? (Probe for level of disclosure and social support/acceptance by network, extent of contribution to household, etc.) |
| --- | --- |
| **Proposed**  **Program** | 1. We are developing a program in which we train women who have been screened and treated for cervical lesions to advocate about reducing cervical cancer risk to other women whom they know. The program will involve meeting with a group of other women who have been screened and treated for cervical cancer lesions for about 2 hours, 6 times over the course of 4 months. The meetings will be dynamic and involve sharing of experiences, role playing how to encourage others to engage in protective behaviors, and activities to promote group unity. What are your initial thoughts about such a program? Would you be interested in hearing more or attending this kind of a group? Why or why not? Do you think other women who have been screened and treated would be interested in attending such a program? Why or why not? 2. What topics would you want or not want to be covered in a program like this? 3. What kinds of activities do you think would be helpful to engage people in the program and motivate and empower them to advocate to other women? 4. Would you be willing to give a testimony or recount personal stories about prevention advocacy, such as using condoms, being screened, or getting treatment, to the group as part of the program? 5. How do you feel about infusing music, singing and dance into the intervention activities as a way to build group solidarity? 6. What kind of person do you think should lead the focus groups? Should it be a peer who is a woman who has been screened and treated for cervical cancer, a professional counselor, or some other type of person? What qualities do you think are important for a facilitator to have? 7. I’d like you know about how you think the group should be scheduled. Should it be every week or every other week? Do you think that people would stay for 2-3 hours, or should it be shorter? 8. Should the groups be separated by age (with younger and older people separate), or can it have a wide range of ages? Why? 9. Can you think of any other suggestions for our program? |

Game Changers: Social Network Member Focus Group Protocol

Notes: Record eligibility information and go through informed consent prior to starting focus group.

Thank you for coming here today. As I mentioned to you when you arrived, Buyinja Health Centre IV is working on a program to help people women who are at risk for developing cervical cancer educate people who they know (their family, friends, and others) about cervical cancer risk. You are being asked to participate in a group discussion to help design this program.

First I’d like to make sure we all agree on some ground rules:

Everyone should participate

One person speaks at a time

No side conversations

Ask if you don’t understand a question

No right or wrong answers

Answer honestly

Respect each other’s opinions

No cell phones/texting

Speak up for recording

*Does anyone have any questions before we begin?*

| **Topic** | **Question(s)** |
| --- | --- |
| Grand Tour | 1. In general, how much do you talk with other women about getting tested for cervical cancer? How about treatment for cervical lesions/treatment after being screened? |

| **Conversations about cervical cancer screening and treatment** | 1. Who do you talk to about cervical cancer screening? What about getting treatment for cervical lesions? (Probe for friends, other women in the community, women in your family, et al.) 2. What topics related to cervical cancer screening and treatment do you talk about? (probe for women’s gynecological health in general, risk reduction, testing, follow-up treatment) 3. Do you ever initiate these conversations about risk for cervical cancer? If so, why, and how do you decide whether to initiate these conversations? How do you start the conversation? With whom do you tend to initiate these conversations? How do you know when someone is open to hearing about cervical cancer screening? How about cervical cancer treatment? 4. [As a follow-up] Please tell me about a recent time when you initiated a conversation about cervical cancer screening; tell me who you talked with, what made you decide to initiate the conversation, what was said, and how the other person reacted. 5. Do other people ever initiate these conversations about cervical cancer screening with you? Do some people you know tend to initiate conversations more than others and if so, who? What kinds of questions do people ask you? (Probe for participant vs. specific others listed) 6. How do you react when other people talk to you about cervical cancer screening? How about treatment after screening? What kind of conversations do you think might lead you to change your attitudes around cervical cancer? Are some kinds of conversations more influential than others on your attitudes or behaviors? 7. What kinds of fears or concerns do you have about talking with other people you know about cervical cancer screening? How about treatment? (Probe for internalized stigma, self-acceptance of cervical cancer risk been screened and treated for cervical lesions to, experiences with in-network discrimination, level of disclosure, etc.) 8. What kinds of things motivate you or make you more comfortable talking with other people about screening? How about treatment? (Probe for level of disclosure and social support/acceptance by network, extent of contribution to household, etc.) |
| --- | --- |
| **Proposed**  **Program** | 1. We are developing a program in which we train women who have been screened and treated for cervical lesions to advocate about reducing cervical cancer risk to other women whom they know. The program will involve meeting with a group of other women who have been screened and treated for cervical cancer lesions for about 2 hours, 6 times over the course of 4 months. The meetings will be dynamic and involve sharing of experiences, role playing how to encourage others to engage in protective behaviors, and activities to promote group unity. What are your initial thoughts about such a program? Would you be interested in hearing more or attending this kind of a group? Why or why not? Do you think women who have been screened and treated would be interested in attending such a program? Why or why not? 2. What are your initial thoughts about such a program? Do you think that women you know who have been screened and treated for cervical lesions would you be interested in hearing more or attending this kind of a group? Why or why not? 3. If you were approached by someone you knew in the program to talk about screening and treatment, how would you react? 4. Can you think of any other suggestions for our program? |

*Note: Questions are a guide and are not meant to be asked verbatim. Additional probes may be used for clarification and questions/topics may be omitted or asked out of order, depending on flow of discussion.*
